# Editorial: Health benefits of flavonoids in diabetes and obesity: from experimental approaches to clinical use

**DOI:** 10.3389/fnut.2023.1312635

**Published:** 2023-10-30

**Authors:** Ali Chaari, Dietrich Büsselberg, Nabil Miled

**Affiliations:** ^1^Weill Cornell Medicine-Qatar, Qatar Foundation, Doha, Qatar; ^2^College of Science, University of Jeddah, Jeddah, Saudi Arabia

**Keywords:** obesity, diabetes, therapy, polyphenols, prevention medicine

Obesity and diabetes are an increasing threat to human health worldwide. These diseases' social and economic impacts have driven researchers to focus on developing more efficient drugs with the lowest drawbacks. This Research Topic choice is to be explained by the interconnections between obesity and diabetes and their tight link to carbohydrates and lipids, digestion, and metabolism. As natural compounds present in essential amounts in vegetables and fruits, phytochemicals represent an attractive alternative to chemical drugs presenting variable toxicities. Among the most characterized phytochemicals, flavonoids have shown high biological activities such as antioxidant, anti-microbial, anti-hypertension, anti-cancer, anti-obesity, and anti-diabetes properties. This Research Topic focuses on the use of flavonoids in the development of potential anti-obesity and anti-diabetes drugs. Research Topics covered the various steps of natural compounds' extraction and characterization, *in vitro* activities, and clinic applications for treating obesity and diabetes.

Research papers in this Research Topic addressed mainly the role of flavonoids in the prevention and control of diabetes and obesity with a particular focus on the already known and possible mechanisms and the mode of action of these phytochemicals. They attempted to identify molecular targets of flavonoids in the various metabolic pathways to support clinical research toward developing future drugs.

Although the biological activities of plant extracts and their anti-obesity and anti-diabetes actions have been extensively studied in the last decades, innovative research focused on *in vivo* studies might foster the development of new drugs with low toxicity and cost.

The research paper entitled “*Body weight change, insulin sensitivity, and adiposity in high-fat (HF) fed rats*” (Alkhalidy et al.) focused on the effects of *Annona squamosa* L. aqueous leaf extract (ASE) on the body weight, and pancreatic insulin content in high-fat fed rats. This research demonstrated that oral administration of a low dose of ASE to HF diet-fed rats significantly reduced the long-term food intake and body weight gain concomitantly, significantly improving insulin sensitivity and reducing fasting blood glucose. Meanwhile, the high dose of ASE displayed a short-term effect on body weight gain and food intake, and in the long term, it improved fasting blood glucose. These findings were in line with the extract's high content in phenolics and flavonoids.

The research paper entitled “*Association between flavonoid and subclasses intake and metabolic associated fatty liver disease in U.S. adults: Results from National Health and Nutrition Examination Survey 2017–2018*” (Tong et al.) assessed the relationship between flavonoid intake and Metabolic associated fatty liver diseases (MAFLD) prevalence in the U.S. adult population through examining the data of 4,431 participants. In this cross-sectional study obtained from National Health and Nutrition Examination Survey between 2017 and 2018, the authors showed that MAFLD was linked with flavonoid subclasses, anthocyanin, and isoflavone. In fact, the study provided evidence that higher anthocyanin and isoflavone intake could prevent MAFLD in the U.S. adult population. The findings of this paper encourage further investigation into the connections and mechanisms between anthocyanin and isoflavone consumption and MAFLD.

In a clinical study with more than 3,000 participants, it was impressively demonstrated that obesity might occur with a BMI that is considered normal. The study is entitled: “*The paradox of obesity with normal weight; a cross-sectional study*” (Lahav et al.). It demonstrated that higher adiposity is associated with an elevated cardiometabolic risk, even for people with normal body weight. Overall, 26% of males and 38% of females with normal BMI may still have excess body fat, which puts them at higher risk, indicating that such factors must be monitored carefully and demonstrate the importance of a healthy diet.

The research paper entitled “*Therapeutic potential of multifunctional myricetin for treatment of type 2 diabetes mellitus*” (Niisato and Marunaka) focused on the relationship between the flavonoid myricetin and the prevalence of type 2 diabetes mellitus (T2DM), emphasizing the significance of incorporating flavonoids into Chinese population's diet to potentially slow the progression or aid in the treatment of T2DM. Although the authors discussed how myricetin possesses several anti-diabetic effects through different mechanisms, they claimed several issues that need to be clarified for the clinical application of myricetin in treating T2DM. In conclusion, a substantial body of evidence drawn from various sources, including epidemiological studies, animal research, and *in vitro* studies, has consistently affirmed the positive impacts of dietary flavonoids containing myricetin on treating and managing T2DM and its related complications. In a review entitled “*Role of flavonoids in controlling obesity: molecular targets and mechanisms*” (Mahboob et al.), potential molecular targets for treating obesity were detailed. These included mainly digestive enzymes, the hormonal system, and gut microbiota. Increasing evidence points to the tight relationship between gut microbiota and metabolic disorders. Emphasis was made on the role of flavonoids in reducing risks of obesity through stimulating diversity and health benefits of gut microbiota. Understanding the molecular mechanisms driving the flavonoid anti-obesity effects was the main objective of this review. The anti-obesity effects of flavonoids were decorticated by detailing their potential effects on digestion, carbohydrate and lipid digestion and metabolism, hormonal system, inflammation, and oxidation. The bioavailability and pharmacokinetics of flavonoids were discussed from the perspective of potential anti-obesity drugs. Tentative of using flavonoids as scaffolds for the structure-based anti-obesity drug design was discussed as an emerging strategy possibly leading to the generation of efficient anti-obesity agents. This review highlighted the potential of flavonoids as promising biomolecules in preventing and treating obesity.

## Author contributions

AC: Conceptualization, Data curation, Formal analysis, Funding acquisition, Investigation, Methodology, Project administration, Resources, Software, Supervision, Validation, Visualization, Writing—original draft, Writing—review & editing. DB: Conceptualization, Data curation, Formal analysis, Funding acquisition, Investigation, Methodology, Project administration, Resources, Software, Supervision, Validation, Visualization, Writing—original draft, Writing—review & editing. NM: Conceptualization, Data curation, Formal analysis, Funding acquisition, Investigation, Methodology, Project administration, Resources, Software, Supervision, Validation, Visualization, Writing—original draft.

